# Defect-free and crystallinity-preserving ductile deformation in semiconducting Ag_2_S

**DOI:** 10.1038/s41598-022-24004-z

**Published:** 2022-11-14

**Authors:** Masaaki Misawa, Hinata Hokyo, Shogo Fukushima, Kohei Shimamura, Akihide Koura, Fuyuki Shimojo, Rajiv K. Kalia, Aiichiro Nakano, Priya Vashishta

**Affiliations:** 1 Faculty of Natural Science and Technology, Okayama University, Okayama, 700-8530 Japan; 2 Department of Physics, Kumamoto University, Kumamoto, 860-8555 Japan; 3 Collaboratory for Advanced Computing and Simulations, Department of Physics and Astronomy, Department of Computer Science, Department of Chemical Engineering and Materials Science, and Department of Biological Science, University of Southern California, Los Angeles, CA 90089-0242 USA

**Keywords:** Mechanical properties, Condensed-matter physics, Structural properties

## Abstract

Typical ductile materials are metals, which deform by the motion of defects like dislocations in association with non-directional metallic bonds. Unfortunately, this textbook mechanism does not operate in most inorganic semiconductors at ambient temperature, thus severely limiting the development of much-needed flexible electronic devices. We found a shear-deformation mechanism in a recently discovered ductile semiconductor, monoclinic-silver sulfide (Ag_2_S), which is defect-free, omni-directional, and preserving perfect crystallinity. Our first-principles molecular dynamics simulations elucidate the ductile deformation mechanism in monoclinic-Ag_2_S under six types of shear systems. Planer mass movement of sulfur atoms plays an important role for the remarkable structural recovery of sulfur-sublattice. This in turn arises from a distinctively high symmetry of the anion-sublattice in Ag_2_S, which is not seen in other brittle silver chalcogenides. Such mechanistic and lattice-symmetric understanding provides a guideline for designing even higher-performance ductile inorganic semiconductors.

## Introduction

With technological development in electronics, semiconductor materials that combine excellent electronic property and mechanical flexibility are desired for next-generation flexible electronics or wearable devices. Currently, organic semiconductor materials are commonly utilized for flexible devices, and technologies for controlling the physical properties and low-cost and low-environmental impact manufacturing processes of its are actively researched^1^. However, their some properties, such as carrier mobility and thermochemical stability, are intrinsically inferior compared to inorganic semiconductors, limiting range of industrial application. Therefore, designing of a new inorganic semiconductor material that has excellent mechanical flexibility is of great importance.

**Figure 1:**
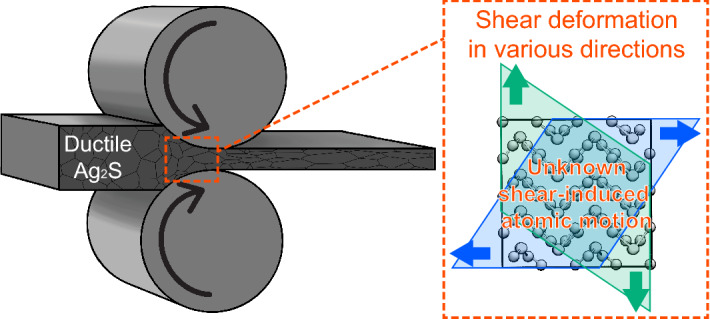
Schematic illustration of research objective. Rolling process of ductile semiconducting Ag_2_S (left) and a shear strained single-crystalline domain (right).

Among inorganic semiconductors, silver sulfide (Ag_2_S) is receiving a lot of attention as a key material toward next-generation flexible devices^2–11^. There are several phases of crystalline Ag_2_S, including a high-temperature (> 450 K) cubic phase (c-Ag_2_S) that exhibits superionic conduction, and a low-temperature monoclinic phase (m-Ag_2_S), which is a semiconductor^12–14^. Most of inorganic semiconductor materials require considerably high temperature to undergo brittle-to-ductile transition^15–18^, but m-Ag_2_S exhibit nevertheless unusual metal-like ductility at room temperature^19^. This unique mechanical property allows production of thin m-Ag_2_S sheet easily by rolling process (left panel in Fig. 1), making m-Ag_2_S a unique semiconductor material for flexible-device applications. In addition of this, their non-toxicity is also a desired property for practical application of semiconductor materials to wearable devices^20–22^. Since no other non-toxicity inorganic semiconductor with high ductility has been found, m-Ag_2_S is of high utility even if it is relatively rare and high-cost among metal sulfides. On the other hand, silver selenide (Ag_2_Se) and silver telluride (Ag_2_Te), which also belong to the silver chalcogenide family and exhibit better electronic properties than Ag_2_S, do not have capability of ductile deformation at room temperature. The drastically different plastic-deformation behaviors among these apparently similar materials suggest distinct atomistic mechanisms. However, a comprehensive explanation of the atomistic behavior under shear deformation in various directions has remain elusive (right panel in Fig. 1). Accordingly, elucidation of atomistic origin of ductility in m-Ag_2_S is necessary to generate an innovative inorganic semiconductor that combines excellent electronic and mechanical properties.

In this study, plastic deformation behaviors of m-Ag_2_S under simple shear deformation has been atomistically investigated using density functional theory based first-principles molecular dynamics (FPMD) simulations. The (100)[010], (100)[001], (010)[100], (010)[001], (001)[100], and (001)[010] shear deformations, where the (*KLM*)[*klm*] shear system means that the (*KLM*) plane is slipped toward [*klm*] direction (Supplementary Fig. S1), were performed and the atomistic behavior through the deformation was analyzed. According to results of the FPMD simulations, we found an omni-directional ductile deformation mechanism in m-Ag_2_S originated from unique symmetricity of the sulfur-sublattice and high mobility of the silver atoms. In this mechanism, dislocation generated by shear loading was immediately annihilated, and their crystallinity was preserved. It is considered that this structural recovering behavior enables m-Ag_2_S to do metal-like ductile deformation. In addition of that, classical MD simulation demonstrate that the structural recovering behavior occurs even for larger system, showing robustness of the proposed ductile deformation mechanism. Furthermore, it was also revealed that Ag_2_Se, which have selenium-sublattice with lower symmetricity, undergoes brittle deformation under simple shear. Our findings will provide a new guideline for atomic level bottom-up design of next-generation flexible inorganic semiconductor materials.

## Results and discussion

**Figure 2:**
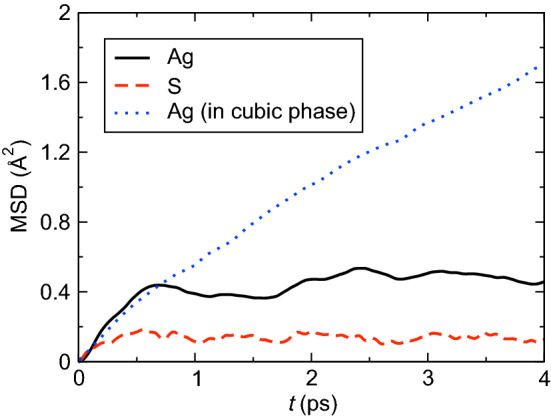
Calculated mean squared displacements (MSDs). MSD of Ag (black solid curve) and S (red dashed curve) ions as a function of time under ambient conditions. The blue dotted curve shows the MSD of Ag ion in cubic superionic conductor phase^23^.

To study structural changes of Ag_2_S under shear strain, we focus on the deformation of the sulfur-sublattice, because thermal fluctuation of Ag atoms in the crystalline m-Ag_2_S is quite large. Figure 2 shows the mean squared displacements (MSDs) of Ag and S atoms in the m-Ag_2_S (black solid and red dashed curves, respectively) and Ag atoms in the c-Ag_2_S (blue dotted curve)^23^ under ambient conditions. While Ag and S atoms in m-Ag_2_S are not diffusing unlike Ag atoms in c-Ag_2_S, the MSD of Ag atoms is > 2.5 times higher than that of S ions, making it difficult to analyze the structure and dynamics of Ag atoms.

**Figure 3:**
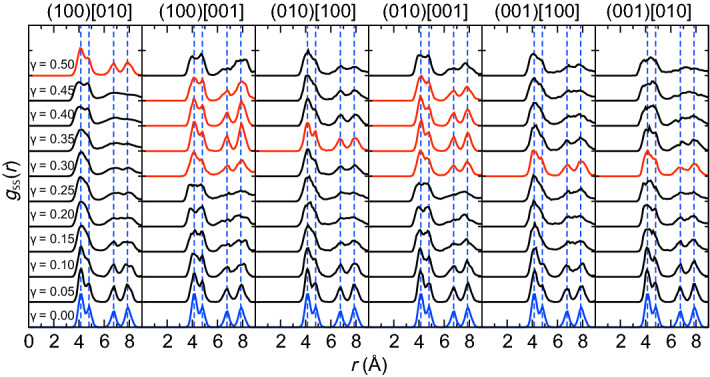
Partial radial distribution functions *g*_SS_(*r*) of sulfur-sublattice during shear deformation. The blue solid curve and dashed line indicates the *g*_SS_(*r*) of non-sheared state and its peak positions, respectively. The red curves shows at which the peak positions were recovered. The *g*_SS_(*r*) was calculated using latest 500 MD step (= 0.5 ps) for each *γ*.

To investigate the structural change of the sulfur-sublattice, the partial pair distribution functions *g*_*αβ*_(*r*) between sulfur atoms *g*_SS_(*r*) were calculated for each shear condition (Fig. 3). The *g*_SS_(*r*) of perfect crystalline m-Ag_2_S (shown in blue solid curves) have four characteristic peaks within 8.0 Å as shown with blue dashed line. Under small shear deformation, those peaks merged, but they reappeared at higher shear in all shear systems as shown with red curves in Fig. 3. The restoration of the characteristic peaks indicates the recovery of the original crystalline structure of the sulfur-sublattice under high shear. This result suggests crystallinity-preserving deformation under large shear strain, which is an origin of its excellent ductility. In contrast to *g*_SS_(*r*), no significant change is seen in the *g*_AgAg_(*r*) and g_AgS_(*r*) during shear deformation (Supplementary Fig. S2).

**Figure 4:**
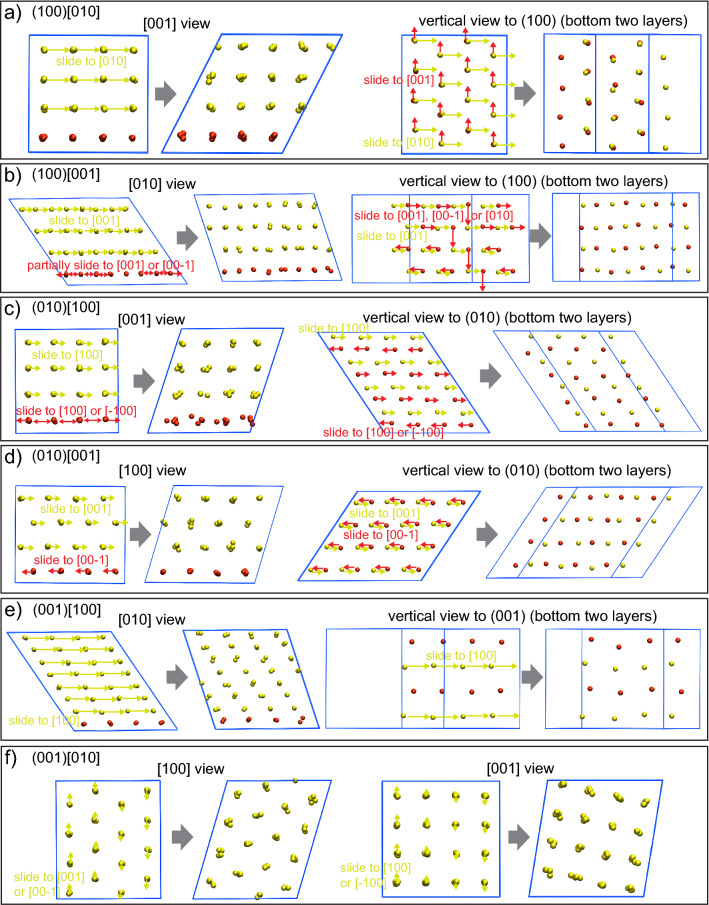
Structural behaviors of m-Ag_2_S under shear deformations. The initial (left side of the gray arrow) and recovered (right side of the gray arrow) atomic configuration in (**a**) (100)[010], (**b**) (100)[001], (**c**) (010)[100], (**d**) (010)[001], (**e**) (001)[100], and (**f**) (001)[010] shear system. The left half shows view from the direction that parallel to the shear plane and perpendicular to the shear direction, and the right half shows view from another direction. Both yellow and red balls indicate sulfur atoms but are classified by their motion.

To examine the mechanisms of the crystallinity-preserving shear deformation, Fig. 4 summarizes the essential atomic motions involved in the structural recovering of sulfur-sublattice (corresponding movies are supplied as Supplementary Videos S1–S6). In the (100)[010], (100)[001], (010)[100], and (010)[001] shear, three quarters of sulfur atoms (yellow spheres in Fig. 4) slid by a half period of the sublattice in the shear direction, while the other quarter (red spheres) behaved differently: (i) (100)[010]: atoms slid by a half period of the sublattice along [001] direction, which is parallel to the shear plane and perpendicular to the shear direction (Fig. 4a); (ii) (100)[001]: atoms slid in (100) plane, which is parallel to the shear plane, but the slide direction is random (Fig. 4b); (iii) (010)[100]: atoms slid along the shear direction not only in the forward direction but also reversely (Fig. 4c); and (iv) (010)[001]: atoms slid to inverse direction of the shear (Fig. 4d). The atomic motion in the (001)[100] shear system (Fig. 4e) is simpler than the above: The 7/8 of the sulfur atoms (yellow spheres in Fig. 4e) slid by a period of the sublattice in the shear direction, while the other atoms (red spheres) kept their position in the supercell. Finally, in the (001)[010] shear system (Fig. 4f), the atoms slid along [001] and [100] directions (not only in the forward but also reverse direction), resulting in axial tilt of the sublattice with respect to the sheared simulation cell. As a result of these atomic motions, the atomic arrangement of the original sulfur-sublattice was restored. What is common in the atomistic behaviors observed in the six shear systems is that structural recovery of sulfur-sublattice is achieved by mass movement of atoms in parallel with a particular plane. Specifically, in the (100)[010], (100)[001], (010)[100], (010)[001], (001)[100], and (001)[010] shear systems, mass movement occurs in parallel with the (100), (100), (010), (010), (001), and (010) plane, respectively. This mechanism is defects-free, resulting in the crystallinity-preserving deformation.

**Figure 5:**
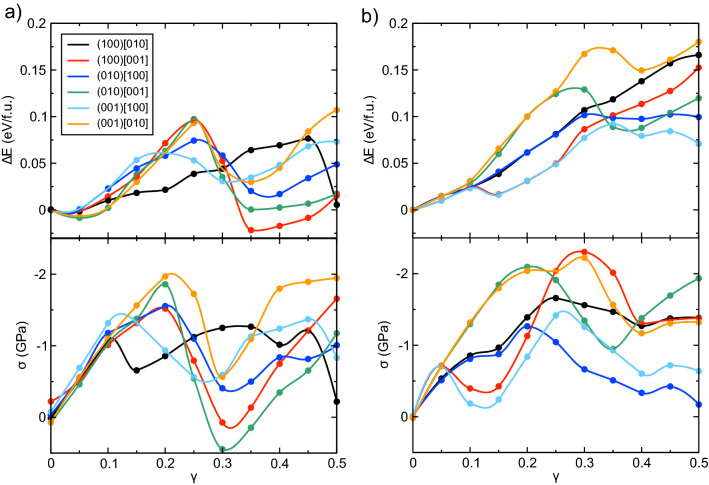
Average potential energy and shear stress. Changes of the average potential energy Δ*E* (upper panel) and average shear stress σ (lower panel) of (**a**) Ag_2_S and (**b**) Ag_2_Se as a function of shear deformation *γ*. The black, red, blue, green, cyan, and orange correspond to the (100)[010], (100)[001], (010)[100], (010)[001], (001)[100], and (001)[010] shear system, respectively. The average value was calculated using latest 500 MD step (= 0.5 ps) for each *γ*. The data points are interpolated using Akima spline method.

If the structural recovering mechanism discussed above is the origin of the excellent ductility in Ag_2_S, this mechanism should not operate in other silver chalcogenides such as Ag_2_Se. To test this hypothesis, FPMD simulations were also performed for Ag_2_Se in the six shear systems with the shear rate of 5.0 × 10^10^ s^−1^. In these Ag_2_Se simulations, the characteristic peaks in *g*_*αβ*_(*r*) were not restored in sharp contrast to Ag_2_S (Supplementary Fig. S2). In addition, we compare changes in the average potential energy and average shear stress as a function of shear deformation between Ag_2_S and Ag_2_Se (Fig. 5). In the Ag_2_S systems, the potential energy increased initially with increasing shear deformation, then exhibited shar drop, before increasing again (upper panel of Fig. 5a). Almost the same trend is seen also in the shear stress (lower panel of Fig. 5b), and the *γ* values for the minimum energy are consistent with those at which the structural recovering occurred (red curves in Fig. 3). On the other hand, in the Ag_2_Se systems, the energy shows an upward trend during the shear deformation (upper panel of Fig. 5b) even if the shear stress was decreasing (lower panel of Fig. 5b). This result indicates that the structure of Ag_2_Se was not recovered but fractured by shear strain.

**Figure 6:**
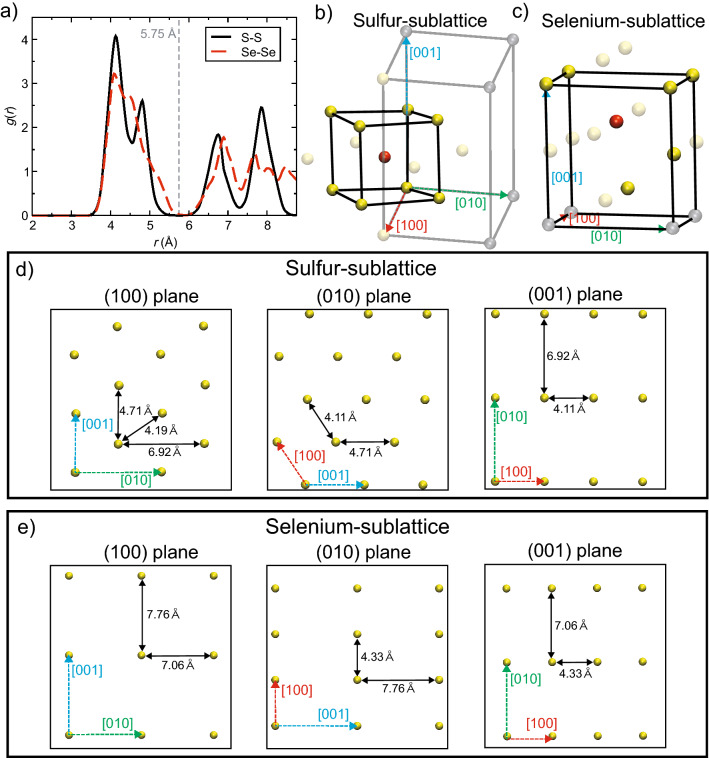
Structural identification of the anion-sublattice in Ag_2_S and Ag_2_Se. (**a**) pair distribution function *g*_SS_(*r*) and *g*_SeSe_(*r*) (red dashed curve) at ambient condition. (**b** and **c**) local geometry around an atom in (**b**) sulfur- and (**c**) selenium-sublattice. The red, yellow and gray balls show the centering, neighbor, and the other atoms, respectively. The black and gray solid lines show unit cell of the anion-sublattices and m-Ag_2_S crystal, respectively, where the unit cell of m-Ag_2_Se crystal is congruent with that of selenium-sublattice. The transparent balls are out of the unit cell. (**d** and **e**) Atomic arrangement and average interatomic distances on the (100) (left), (010) (middle), and (001) (right) plane in (**d**) sulfur- and (**e**) selenium-sublattice. The red, green, and blue dashed arrows in b to e show [100], [010], and [001] direction, respectively.

To understand the reason why the structural recovering mechanism works only in m-Ag_2_S but not in Ag_2_Se, we compared local geometries of the sulfur- and selenium-sublattices (Fig. 6). To clarify the neighboring atoms of a sulfur and selenium atom in their sublattice, cutoff interatomic distance is determined as 5.75 Å based on the *g*_SS_(*r*) and g_SeSe_(*r*) under ambient conditions (Fig. 6a). Using this criterion, it was confirmed that both sulfur and selenium atoms have 14 neighboring atoms in their sublattice (Figs. 6b and c). We can see that the unit structure of sulfur-sublattice, which is smaller than the crystalline unit cell of m-Ag_2_S (gray frame in Fig. 6b) and forms BCC-like structure with > 5 Å on each side, is entirely contained in this neighborhood (black solid frame in Fig. 6b). In contrast, the unit structure of selenium sublattice protrudes from the neighborhood, and has much lower symmetry than sulfur-sublattice (black solid frame in Fig. 6c). These differences in size of the unit structure and geometrical symmetry strongly influence deformation behaviors. To realize the mass movement of atoms necessary for the structural recovery, atoms in the anion-sublattice need be linearly arranged at nearly equal intervals along a crystal axis direction.

In the sulfur-sublattice, average interval distance of sulfur atoms along [100], [010], and [001] direction is 4.11, 6.92, and 4.71 Å, respectively, making it easy to operate the mass movement of sulfur atoms. The interval distance along [010] direction is relatively long, but there is a shorter pathway to move on (100) plane, which is along [011] direction with an interval of 4.19 Å (left panel of Fig. 6d). Indeed, the atomic motion along [011] direction was observed in the (100)[010] shear system. On the other hand, average interval distance of selenium atoms along [100], [010], and [001] direction is 4.33, 7.06, and 7.76 Å, respectively (Fig. 6e). In comparison to sulfur-sublattice, this geometry is not suitable for crystallinity-preserving movement of selenium atoms. This geometrical analysis indicates that the remarkable ductility of m-Ag_2_S originates from the high symmetry of sulfur-sublattice with small unit-cell size. Those findings suggest that symmetry modification of the anion-sublattice by element substituent or impurity doping essentially controls ductile *vs*. brittle behaviors of metal chalcogenides. Meanwhile, element substituent or impurity doping *without* symmetry breaking of the sulfur sublattice allows us to control electronic properties of m-Ag_2_S while maintaining its ductility. This new understanding will thus help develop future high-performance and flexible semiconductor materials. For this purpose, larger spatial/time scale than FPMD and higher accuracy than classical MD is required. With a recent progress of machine-leaning interatomic potentials (MLIPs), such large-scale molecular simulations of metal chalcogenides with high accuracy are becoming feasible^24–28^. We plan to apply advanced computational techniques such as MLIPs for further investigations of Ag_2_S systems.

To show the robustness of the structural recovering mechanism with respect to system sizes and models, we performed classical MD simulations involving a much larger number of atoms (see Supplementary Methods). A simulation cell comprising 262,144 Ag_2_S (786,432 atoms) was employed as the simulation model, in which an empirical interatomic potential was used^29^. While grain boundaries were formed and the relaxation time became much longer than the small FPMD system (Supplementary Fig. S3), the crystallinity-preserving structural recovery were observed under (100)[001] shear (Supplementary Fig. S4). This result observed in classical MD demonstrates robustness of the ductile deformation mechanism proposed by FPMD.

Here, it is worth noting that our defect-free, omni-directional, and crystallinity-preserving deformation mechanism is distinct from previously proposed plastic deformation mechanisms of m-Ag_2_S. Shi et al.^19^ demonstrated that the layers in m-Ag_2_S slip easily based on first-principles calculation. According to their density functional calculation, a layer in m-Ag_2_S slipped along [001] direction by allowing change in the interlayer distance. The calculated activation barrier for layer slipping is significantly lower than those in typical brittle materials. Besides the above pioneering work, Li et al.^30^ studied the structural and bonding properties of m-Ag_2_S under shear and tensile strains. They performed structural relaxation during pure shear deformations and found that shear stress does not lead to failure in two types of shear directions up to the shear deformation of γ = 1.0. Under the shear strain, m-Ag_2_S undergoes plastic deformation instead of fracture accompanied by bond-breaking and bond-recovery behaviors. Those plastic deformation mechanisms are reasonable, nevertheless work only to limited crystallographic directions. From this viewpoint, our omni-directional deformation mechanism is clearly distinct from the previously proposed mechanisms.

Finally, we briefly remark on the role of silver atoms in the structural recovering mechanism. As shown in Fig. 2, silver atoms exhibit higher mobility than sulfur atoms at room temperature. This endows high flexibility to silver-sulfur bonding that constitutes m-Ag_2_S. Due to their higher mobility, silver atoms can readily follow the mass movement of sulfur atoms. This motion in turn is accompanied by frequent switching of chemical bonding, which is afforded by the flexible silver-sulfur bonds. Such flexibility of silver-sulfur bonds was also shown in the previous works^19,30^.

## Computational method

The electronic states are calculated by projector augmented-wave method^31,32^, within a framework of density functional theory (DFT). Plane-wave cutoff energies of 20 and 200 Ry. are employed for wave function and electron density, respectively. Only Γ-point is used for the Brillouin zone sampling. The Perdew-Burke-Ernzerhof exchange correlation energy functional is used^33^. The 4*d*^10^5*s*^1^5*p*^0^, 3*s*^2^3*p*^4^3*d*^0^ and 4*s*^2^4*p*^4^4*d*^0^ orbitals are employed as the valence states of silver, sulfur and selenium atoms, respectively. The DFT + *U* method with *U*_eff_ = 6.0 eV is used for the 4*d*-electrons of silver atoms to correct on-site Coulomb interaction of localized *d*-electrons. It is revealed that such correction is necessary to reproduce the crystal structure and atomistic dynamics accurately in silver chalcogenide systems^34,35^. Additionally, the empirical correction for the van der Waals interaction is taken into account to reproduce the interlayer weak bonding^36,37^. The periodic boundary conditions are applied for all direction.

Supercells consist of 192 atoms (Ag_128_S_64_ or Ag_128_Se_64_). The equations of motions are solved at 300 K under Canonical ensemble with the time step of 1.0 fs. First, simple shear deformations with *γ* = 0.05 are loaded every 1.0 ps, which corresponds to the shear rate *dγ*/*dt* = 5.0 × 10^10^ s^−1^. While this value seems quite large compared with experimental conditions, we note that our previous tensile simulation with the same order of strain rate provides reasonable structural behaviors^38^. The FPMD simulations with the (100)[010], (100)[001], (010)[100], (010)[001], (001)[100], and (001)[010] shear systems of Ag_2_S and Ag_2_Se up to *γ* = 0.50 (= 10 ps) were performed to study the structural behaviors under shear strains, where the (*KLM*)[*klm*] shear system means that the (*KLM*) plane is slipped toward [*klm*] direction during the shear deformation (Supplementary Fig. S1). In the (100)[010] and (010)[001] shear system, structural changes with shear rate *dγ*/*dt* > 6.25 × 10^9^ s^−1^ are additionally examined because the structural recovering behaviors were not observed with *dγ*/*dt* = 5.0 × 10^10^ s^−1^ in only these two shear systems. The detailed simulation schedule is summarized in Supplementary Fig. S5. All FPMD simulations in this study were performed using the QXMD code^39,40^.

## Supplementary Information

Supplementary Information 1.

Supplementary Video 1.

Supplementary Video 2.

Supplementary Video 3.

Supplementary Video 4.

Supplementary Video 5.

Supplementary Video 6.

## Data Availability

All data are available in the main text or the Supplementary Information.
