# Supplementary material for: Defect-free and crystallinity-preserving ductile deformation in semiconducting Ag2S
Source: Sci Rep. 2022 Nov 14;12:19458. doi: 10.1038/s41598-022-24004-z (PMC9663522; doi:10.1038/s41598-022-24004-z)
Supplement: Supplementary file 1 — Supplementary Information 1. [file 41598_2022_24004_MOESM1_ESM.pdf]

Supplementary Information for:

Defect-free and crystallinity-preserving ductile deformation in semiconducting Ag_2S

Masaaki Misawa[1*], Hinata Hokyo[2], Shogo Fukushima[2], Kohei Shimamura[2], Akihide Koura[2], Fuyuki Shimojo[2], Rajiv K. Kalia[3], Aiichiro Nakano[3], and Priya Vashishta[3]

[1]Faculty of Natural Science and Technology, Okayama University, Okayama 700-8530, Japan.

[2]Department of Physics, Kumamoto University, Kumamoto 860-8555, Japan.

[3]Collaboratory for Advanced Computing and Simulations, Department of Physics and Astronomy, Department of Computer Science, Department of Chemical Engineering and Materials Science, and Department of Biological Science, University of Southern California, Los Angeles, CA 90089-0242, USA.

Corresponding author: misawa@okayama-u.ac.jp

Supplementary Methods

To investigate system size effects on the structural recovering behaviors of m-Ag_2S, an additional simulation based on classical MD method was performed with an empirical interatomic potential (EIP) for Ag_2S. The EIP was created on the basis of EIP for Ag_2Se [S1] which is the reliable analytical potential for silver chalcogenides. While the same series of function forms employed in the EIP for Ag_2Se was adopted, all parameters were determined by the parameter scaling method

[S2] to compensate the difference in volume between Ag_2Se and Ag_2S. We confirmed that the monoclinic structure of Ag_2S is satisfactorily retained by this potential. The particle mesh Ewald method is used for calculating long-range Coulomb interaction. A simulation cell consisting of 786,432 atom (16×16×16 expanded system of the 192 atomic Ag_2S system) in which the periodic boundary conditions for all cartesian directions were taken into account was deformed under (100)[001] shear load. The MD simulation was performed with canonical ensemble at the temperature of 300 K, with a timestep of 1 fs. First, the MD simulation under non-share condition was performed for 15,000 steps (15 ps) for relaxation. Subsequently, the (100)[001] shear deformations with $\gamma = 0.05$ are loaded every 5,000 steps (5 ps) up to $\gamma = 0.35$ to reproduce the structural recovering behaviors. Up to $\gamma = 0.30$, no structural recovering behavior was observed. Finally, the potential energy tended to decrease within 5 ps at $\gamma = 0.35$, and the MD simulation continued for another 270,000 steps (270 ps) for relaxation. At $\gamma = 0.35$, which corresponds to the shear strain that gives the structural recovering behavior for the 192-atom FPMD system, massive structural modification accompanied by formation of the grain boundaries (Supplementary Figs. S3(b) and (c)) occurred with gradually decreasing the potential energy (Supplementary Fig. S3(a)). Because the potential energy continues to decrease, the grain boundaries may be eliminated by relaxing the atomic structure with long-term MD simulations. During the relaxation at $\gamma = 0.35$, the first major peak in the partial radial distribution function was recovered (Supplementary Fig. S4), showing that the crystalline structure was recovered except on the grain boundaries. According to the results of the MD simulation, and also considering the fact that the structural recovering behavior begins at the same shear strain as the 192-atom FPMD system, we believe that the same mechanism of structural recovering as observed in the small FPMD system works locally even for realistically large systems.

Supplementary Figures

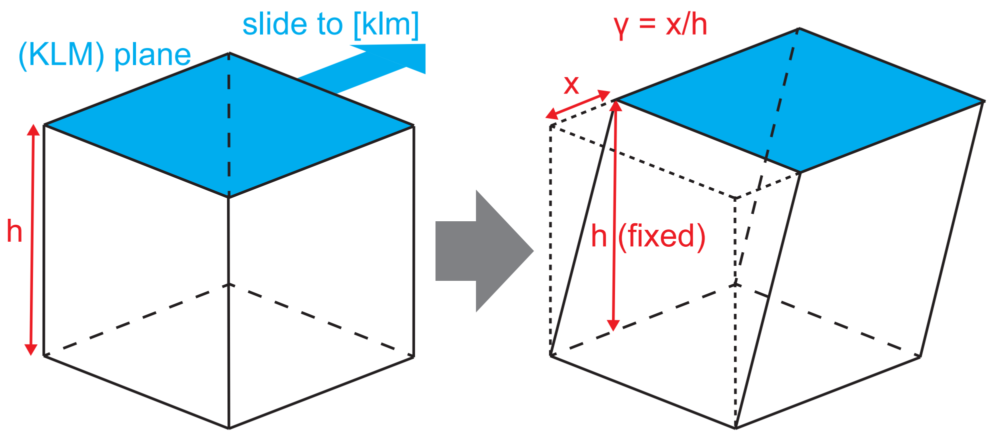

Supplementary Figure S1. Schematic image of simple (*KLM*)[*klm*] shear deformation. The (*KLM*) plane shown as blue area of the box is slide to the [*klm*] direction shown as blue arrow. The h and x are the height of the box and slide distance of the (*KLM*) plane, respectively. During a simple (*KLM*)[*klm*] shear deformation, h and volume of the box are completely preserved. The shear deformation γ is defined as $\gamma = x/h$.

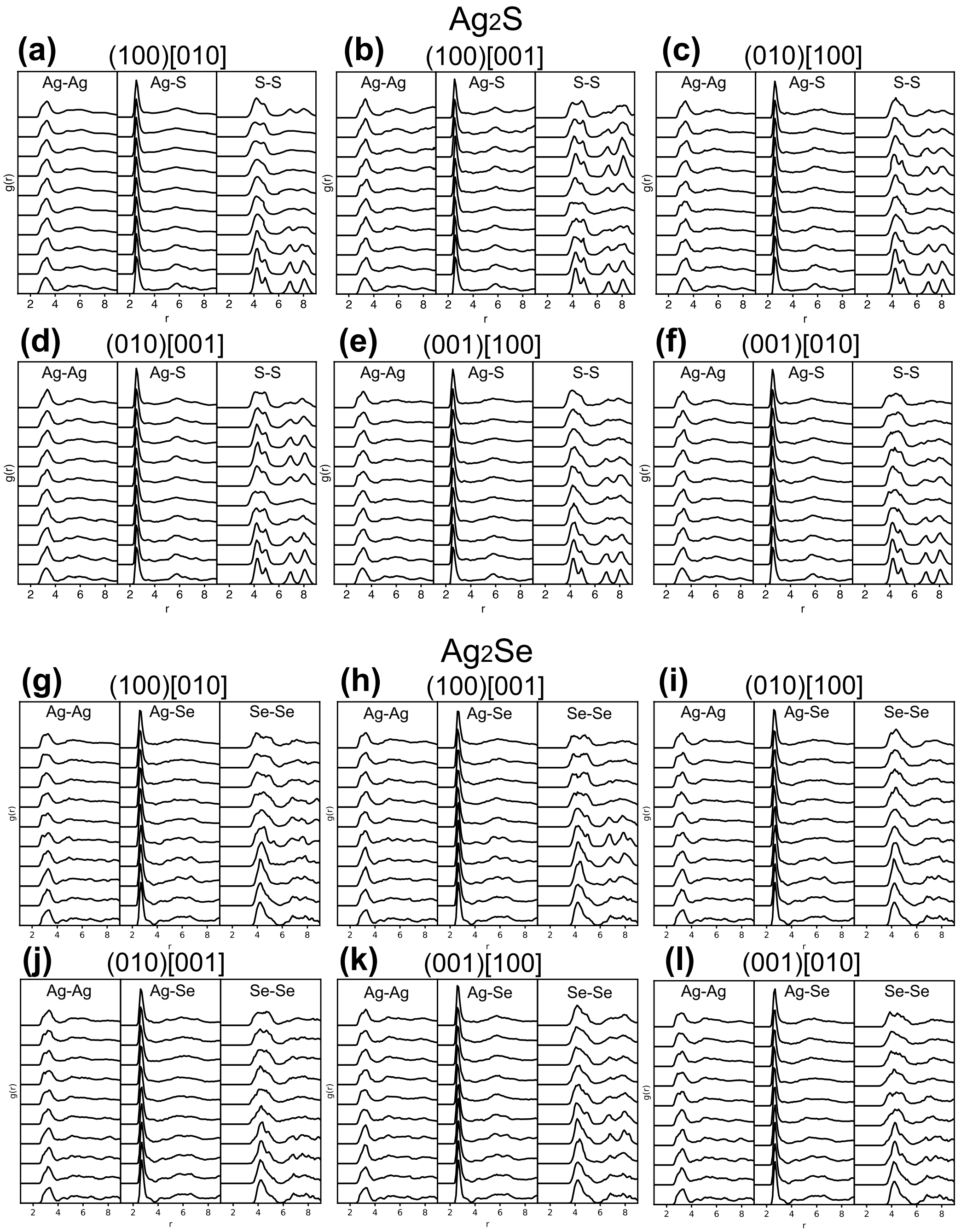

Supplementary Figure S2. Partial pair distribution functions $g_{\alpha\beta}(r)$ (α, β = Ag, S, and Se). $g_{\alpha\beta}(r)$s of Ag_2S ((a) to (f)) and Ag_2Se ((g) to (l)) in (100)[010] ((a) and (g)), (100)[001] ((b) and (h)), (010)[100] ((c) and (i)), (010)[001] ((d) and (j)), (001)[100] ((e) and (k)), and (001)[010] ((f) and (l)) shear system, respectively. The average $g_{\alpha\beta}(r)$s at shear deformation γ = 0, 0.05, 0.10, 0.15, 0.20, 0.25, 0.30, 0.35, 0.40, 0.45, and 0.50 are displayed from the bottom to the top.

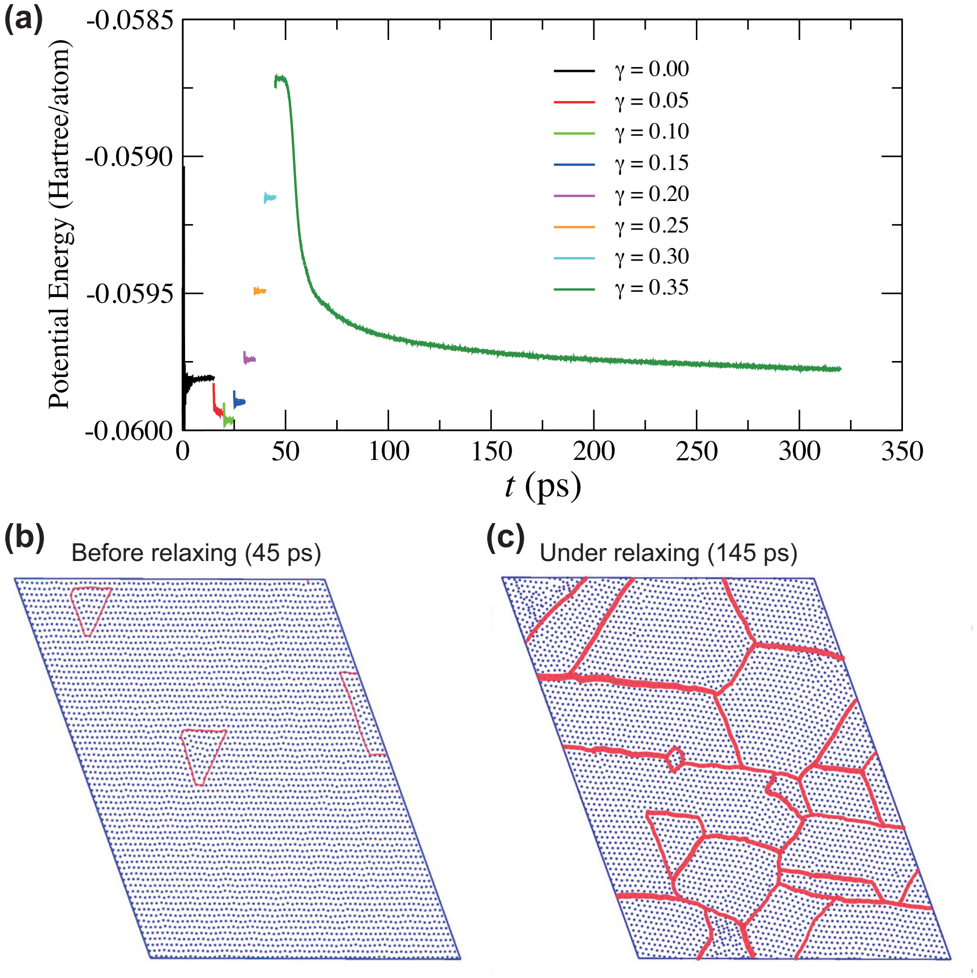

Supplementary Figure S3. Result of the classical MD simulation. Evolution of the potential energy during the MD simulation (a). Observed grain boundaries in the simulation model at 45 ps (b) and 145 ps (c). The blue dots and red lines in (b) and (c) show the anions and grain boundaries, respectively.

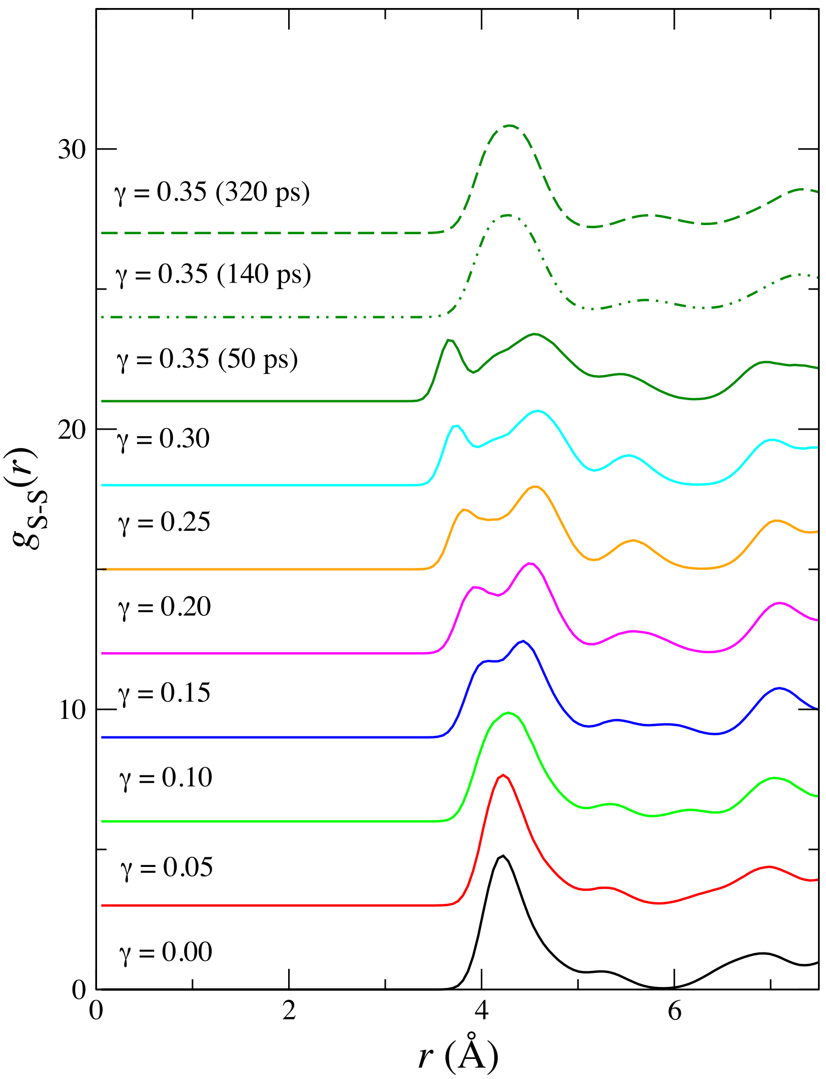

Supplementary Figure S4. Partial pair distribution functions $g_{\alpha\beta}(r)$ ($\alpha = \beta =$ S) obtained by the classical MD simulation. $g_{\text{SS}}(r)$ under (100)[001] shear. The average $g_{\text{SS}}(r)$ s at shear deformation $\gamma = 0$, 0.05, 0.10, 0.15, 0.20, 0.25, 0.30, and 0.35 are displayed from the bottom to the top. The green dashed-dotted and dashed curves show the $g_{\text{SS}}(r)$ s under relaxation at $\gamma = 0.35$.

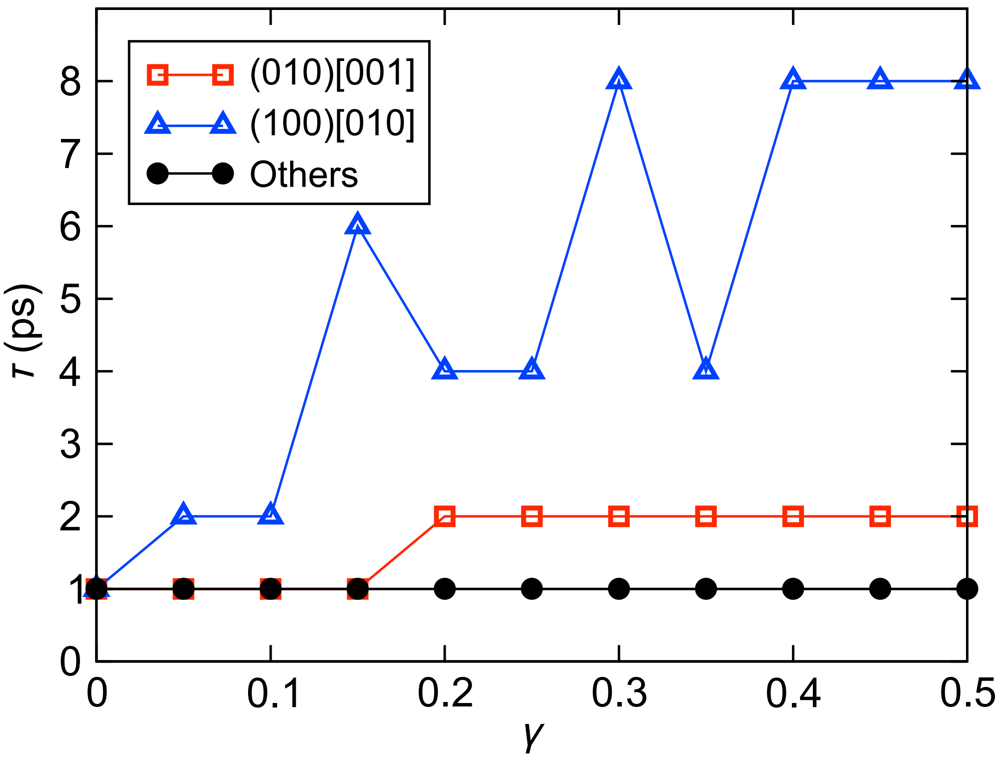

Supplementary Figure S5. Computational schedule of the shear simulations for Ag_2S. The horizontal and vertical axis indicate shear deformation γ and corresponding computational time τ, respectively. The maximum and minimum shear rate $d\gamma/dt$ correspond to 5.0×10^{10} s^{-1} (τ = 1 ps) and 6.25×10^{9} s^{-1} (τ = 8 ps), respectively.

Supplementary Videos:

Supplementary Video S1. the structural recovering in (100)[010] shear system. The direction of view and color of atoms are corresponding to Fig. 5a in the main text.

Supplementary Video S2. the structural recovering in (100)[001] shear system. The direction of view and color of atoms are corresponding to Fig. 5b in the main text.

Supplementary Video S3. the structural recovering in (010)[100] shear system. The direction of view and color of atoms are corresponding to Fig. 5c in the main text.

Supplementary Video S4. the structural recovering in (010)[001] shear system. The direction of view and color of atoms are corresponding to Fig. 5d in the main text.

Supplementary Video S5. the structural recovering in (001)[100] shear system. The direction of view and color of atoms are corresponding to Fig. 5e in the main text.

Supplementary Video S6. the structural recovering in (001)[010] shear system. The direction of view and color of atoms are corresponding to Fig. 5f in the main text.

Supplementary References

[S1] J. P. Rino, Y. M. M. Hornos, G. A. Antonio, I. Ebbsj ̈o, R. K. Kalia, and P. Vashishta, Structural and dynamical correlations in Ag2Se: A molecular dynamics study of superionic and molten phases, *J. Chem. Phys*. **89**, 7542 (1988).

[S2] F. Shimojo and M. Kobayashi, Molecular Dynamics Studies of Molten AgI.I. Structure and Dynamical Properties, *J. Phys. Soc. Jpn.* **60**, 3725 (1991).
